# Supplementary material for: Causal interplay between lactose intolerance and gut microbiota: a combined bidirectional Mendelian randomization and in vivo validation study
Source: Front Nutr. 2026 Jun 1;13:1803337. doi: 10.3389/fnut.2026.1803337 (PMC13265576; doi:10.3389/fnut.2026.1803337)
Supplement: Supplementary file 1 [file Data_Sheet_1.zip › supplementary materials/Forward/leave one out cross validation/ebi-a-GCST90027653.finngen_R12_E4_LACTONAS.pdf]

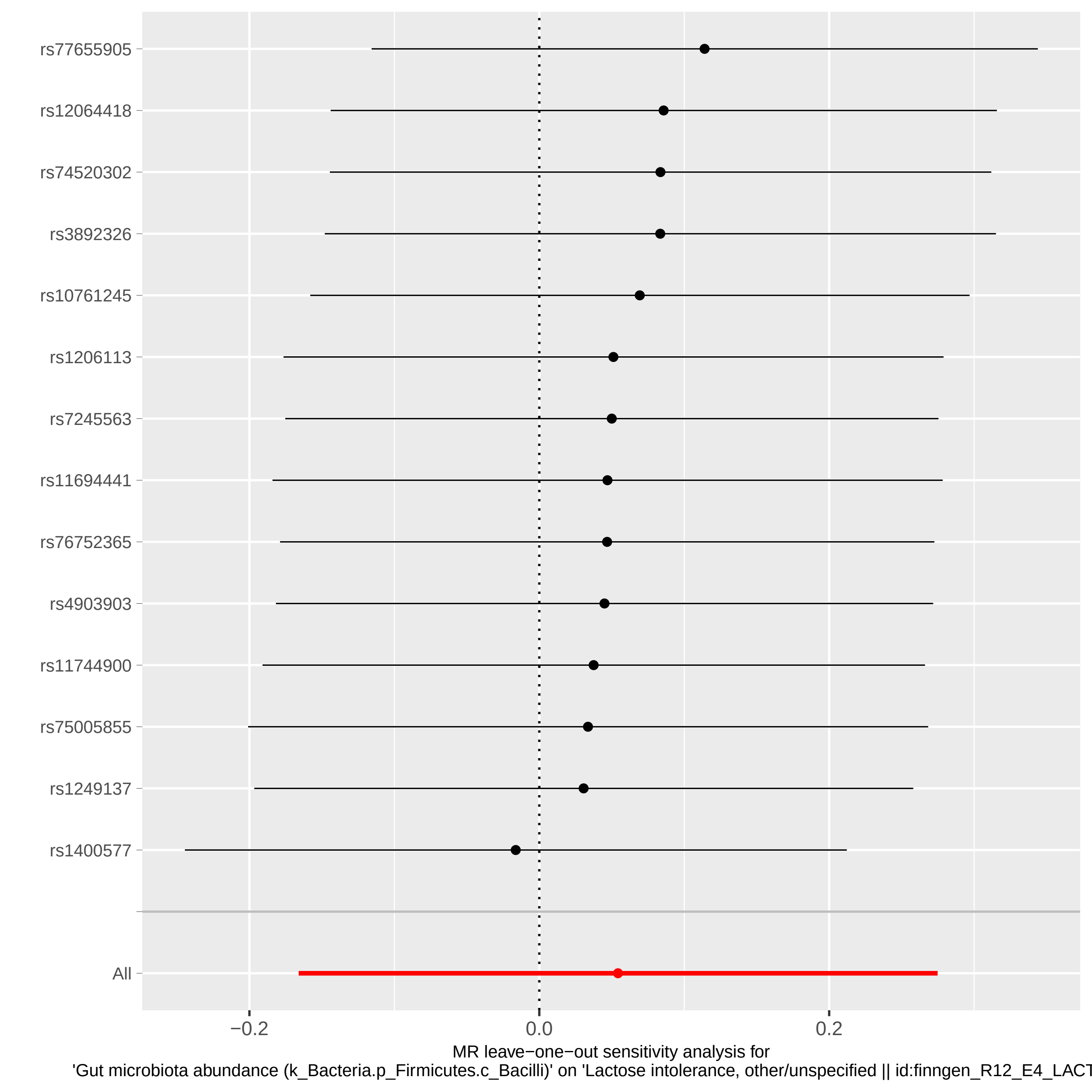

rs77655905
rs12064418
rs74520302
rs3892326
rs10761245
rs1206113
rs7245563
rs11694441
rs76752365
rs4903903
rs11744900
rs75005855
rs1249137
rs1400577
All
−0.2
0.0
0.2
MR leave−one−out sensitivity analysis for
'Gut microbiota abundance (k_Bacteria.p_Firmicutes.c_Bacilli)' on 'Lactose intolerance, other/unspecified || id:finngen_R12_E4_LACT
